# A Lassa virus live attenuated vaccine candidate that is safe and efficacious in guinea pigs

**DOI:** 10.1038/s41541-024-01012-w

**Published:** 2024-11-17

**Authors:** Brian D. Carey, Shuiqing Yu, Jillian Geiger, Chengjin Ye, Louis M. Huzella, Rebecca J. Reeder, Monika Mehta, Shawn Hirsch, Rebecca Bernbaum, Beatrice Cubitt, Bapi Pahar, Scott M. Anthony, Anthony Marketon, John G. Bernbaum, Julie P. Tran, Ian Crozier, Luis Martínez-Sobrido, Gabriella Worwa, Juan Carlos de la Torre, Jens H. Kuhn

**Affiliations:** 1 Integrated Research Facility at Fort Detrick, National Institute of Allergy and Infectious Diseases, National Institutes of Health, Fort Detrick, Frederick, Maryland USA; 2 Department of Disease Intervention and Prevention, Texas Biomedical Research Institute, San Antonio, Texas USA; 3 Department of Immunology and Microbiology, The Scripps Research Institute, La Jolla, California, USA; 4 Clinical Monitoring Research Program Directorate, Frederick National Laboratory for Cancer Research, Frederick, Maryland USA

**Keywords:** Arenaviruses, Viral infection

## Abstract

Lassa virus (LASV) is a rodent-borne mammarenavirus that causes tens to hundreds of thousands of human infections annually in Western Africa. Approximately 20% of these infections progress to Lassa fever (LF), an acute disease with case–fatality rates from ≈20–70%. Currently, there are no approved vaccines or specific therapeutics to prevent or treat LF. The LASV genome consists of a small (S) segment that has two genes, *GP* and *NP*, and a large (L) segment that has two genes, *L* and *Z*. In both segments, the two genes are separated by non-coding intergenic regions (IGRs). Recombinant LASVs (rLASVs), in which the L segment IGR was replaced with the S segment IGR or in which the *GP* gene was codon-deoptimized, lost fitness in vitro, were highly attenuated in vivo, and, when used as vaccines, protected domesticated guinea pigs from otherwise lethal LASV exposure. Here, we report the generation of rLASV/IGR-CD, which includes both determinants of attenuation and further enhances the safety of the vaccine compared with its predecessors. rLASV/IGR-CD grew to high titers in Vero cells, which are approved for human vaccine production, but did not cause signs of disease or pathology in guinea pigs. Importantly, guinea pigs vaccinated with rLASV/IGR-CD were completely protected from disease and death after a typically lethal exposure to wild-type LASV. Our data support the development of rLASV/IGR-CD as a live-attenuated LF vaccine with stringent safety features.

## Introduction

First described in 1970^1^, Lassa virus (LASV; *Arenaviridae*: *Mammarenavirus lassaense*^2^) is estimated to cause tens to hundreds of thousands of human infections per year in Western Africa^3^. LASV is hosted by murid rodents: mainly Natal mastomys (*Mastomys natalensis* Smith, 1834) but also African hylomyscus (*Hylomyscus pamfi* Nicolas, Olayemi, Wendelen & Colyn, 2010), Baoule’s mice (*Mus baoulei* (Vermeiren & Verheyen, 1980)), reddish-white mastomys (*Mastomys erythroleucus* (Temminck, 1853)), and likely others^4–7^. Human infections occur when proximity to infected rodents or their contaminated excreta, secreta, or tissues facilitates exposure to aerosolized LASV. Person-to-person transmission primarily occurs in health-care settings through contact with contaminated bodily fluids or via fomites^8–12^. Approximately 20% of LASV infections progress to Lassa fever (LF), which has high but variable case–fatality rates of ≈20–70%^13–21^. Generally, LF is characterized by an acute onset of a non-specific febrile prodrome (including malaise, arthralgia, myalgia, and gastrointestinal disturbances), followed variably by many other clinical manifestations. Severely ill patients develop multi-system organ dysfunction, including acute kidney injury, central nervous system involvement, and respiratory dysfunction that are often harbingers of impending shock and death. Up to one third of LF survivors develop unilateral or bilateral sensorineural hearing loss; other post-acute sequalae are described but poorly characterized^8,22–27^. Beyond general supportive care, LASV-specific treatment is limited to common off-label use of intravenous or oral ribavirin despite insubstantial evidence of efficacy as well as safety concerns^28–33^. Further, there are no approved vaccines for use as prevention or post-exposure prophylaxis for LF^8,34,35^. Consequently, the World Health Organization (WHO) added LF to the revised list of priority diseases for development of safe and effective vaccines^3^.

LASV has a bisegmented RNA genome and produces enveloped spherical to pleomorphic particles^36^. The independently encapsidated small (S) and large (L) genome segments each direct the synthesis of two proteins using an ambisense coding strategy by two adjacent open reading frames that are separated by non-coding intergenic regions (IGRs)^36^. The S segment encodes a nucleoprotein (NP), which encapsidates viral RNAs and functions as an interferon (IFN) antagonist^37–39^, and a glycoprotein precursor (GPC) that is processed to a glycoprotein (GP) complex. This complex is comprised of subunits GP1 and GP2 and a stable signal peptide (SSP) that together mediate virion cell entry^40–42^. The L segment encodes a large (L) protein with an RNA-directed RNA polymerase (RdRp) domain that mediates viral transcription and replication^36^ and a zinc-binding protein (Z) that mediates virion assembly and budding and also serves as an IFN antagonist^2,36^.

To evaluate the feasibility of creating a live-attenuated vaccine (LAV) against LF, we previously generated two recombinant LASVs by reverse genetics: in one, the L segment IGR was replaced by the S segment IGR (rLASV/IGR(S-S))^43^; in the other, the wild-type *GP* gene, which encodes GPC, was codon-deoptimized (rLASV-GPC/CD)^44^. Both viruses proved to be less fit in vitro and highly attenuated in vivo, and a single subcutaneous injection of each recombinant virus completely protected strain 13 domesticated guinea pigs (*Cavia porcellus* (Linnaeus, 1758)) subcutaneously exposed 30 d later to a typically lethal dose of wild-type (LASV-WT) or domesticated guinea-pig-adapted (GPA-LASV) LASV. In addition, these studies demonstrated that both recombinant viruses were genetically stable during serial passages in cell-culture^43,44^.

The modifications in rLASV were restricted to single genomic segments (i.e., the L segment for rLASV/IGR(S-S) and the S segment for rLASV-GPC/CD). The possibility that these recombinant viruses, if deployed as LAVs, could reassort with endemic non-virulent LASV strains to regain virulence, was of plausible concern. In a proof-of-concept study, a recombinant version of lymphocytic choriomeningitis virus (LCMV), a close genetic relative of LASV, was modified to contain both segment modifications simultaneously (rLCMV/IGR-CD)^45^. As expected, rLCMV/IGR-CD was highly attenuated and yet subsequently completely protected laboratory mice against a typically lethal exposure to recombinant wild-type LCMV (rLCMV-WT). Importantly, rLCMV/IGR-CD prevented the generation of reassortant viruses with increased virulence in coinfection experiments with rLCMV-GPC/CD or rLCMV/IGR(S-S)^45^. These results indicated that mammarenavirus LAVs containing attenuating modifications in both genomic segments, thereby exhibiting an unbreachable attenuated phenotype, could be developed. Here, we report the generation of a rLASV incorporating both attenuating features (rLASV/IGR-CD) and its evaluation as an LAV in two established guinea pig models of LF.

## Results

### Generation and characterization of rLASV/IGR-CD in cultured cells

**Fig. 1:**
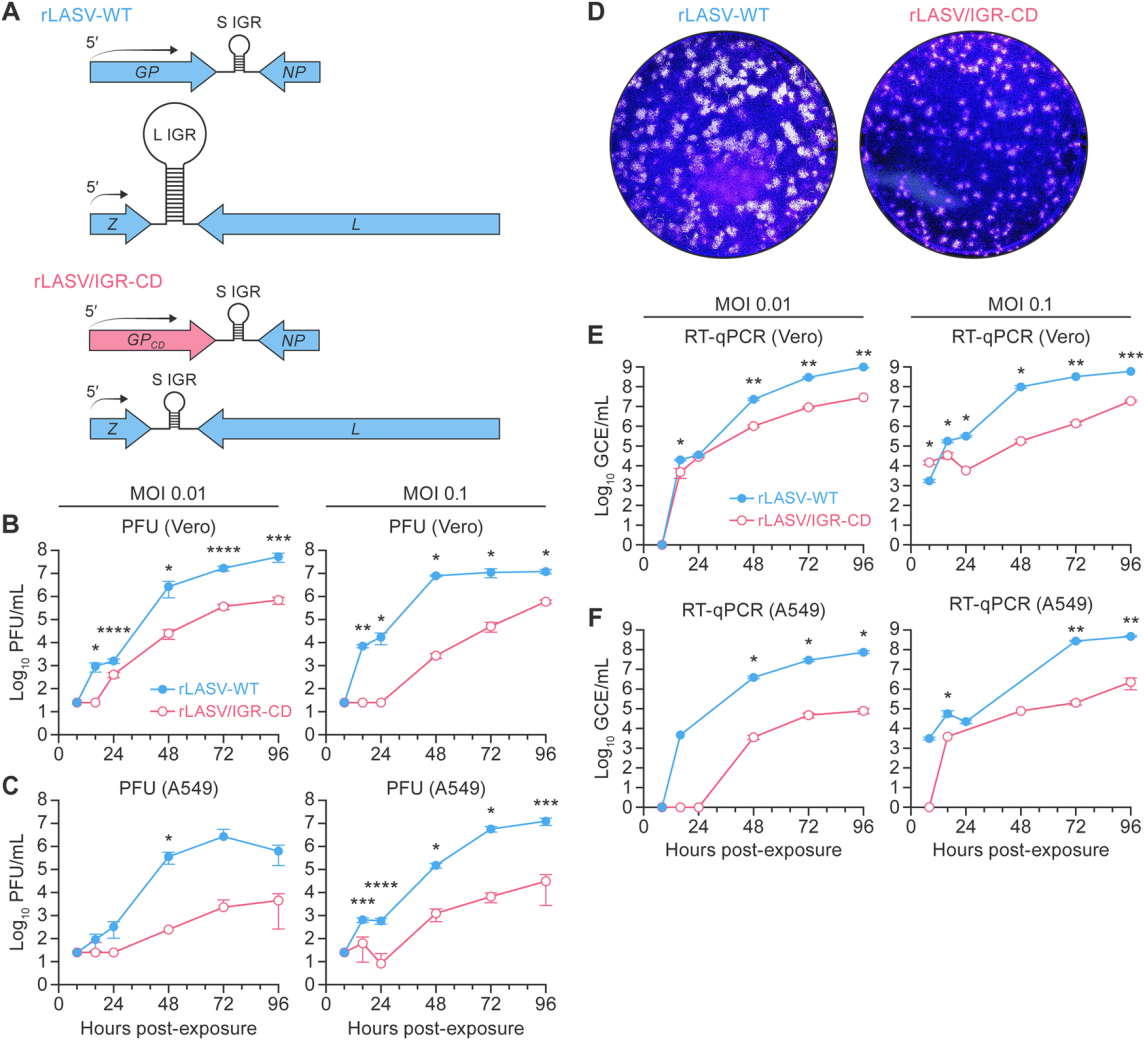
Generation and characterization of rLASV/IGR-CD in cultured cells*.* **A** Schematic of the wild-type LASV genome used to create the recombinant control Lassa virus (rLASV-WT; top) and the locations of attenuating changes in rLASV/IGR-CD (bottom). **B** Vero cells or **C** A549 cells were exposed to rLASV-WT or rLASV/IGR-CD at the indicated MOIs, and supernatants were collected at the indicated time points. Virus titers were measured by plaque assay. **D** Representative images showing the morphology of plaques caused by rLASV-WT and rLASV/IGR-CD in Vero E6 cells. **E** Vero cells or **F** A549 cells were exposed to rLASV-WT or rLASV/IGR-CD at the indicated MOIs, and supernatants were collected at the indicated time points. Viral RNA levels were measured by RT-qPCR. *n* = 3, error bars represent standard deviation. *p < 0.05, **p < 0.005, ***p < 0.0005, ****p < 0.00005. CD codon-deoptimized; GCE genome copy equivalents; *GP* glycoprotein gene; *GP*_*CD*_ codon-deoptimized glycoprotein gene; IGR intergenic region; L large segment; *L* large protein gene; LASV Lassa virus; MOI multiplicity of infection; *NP* nucleoprotein gene; PFU plaque-forming units; rLASV recombinant LASV; RT-qPCR real-time reverse transcription polymerase chain reaction; S small segment; WT wild-type; *Z* zinc-binding protein gene.

rLASV/IGR-CD, a recombinant LASV with a codon-deoptimized *GP* gene (*GP*_*CD*_) and an S segment IGR in place of the L segment IGR (Fig. 1A), was constructed and rescued using the same procedure previously described for rLASV/IGR(S-S)^43^ and rLASV-GPC/CD^44^ (Fig. 1A). In a side-by-side rescue experiment, wild-type LASV (rLASV-WT) plaques were detectable at Day 9 post-transfection, whereas rLASV/IGR-CD plaques were first detectable at Day 13 (Supplementary Fig. 1). Sanger sequencing confirmed the presence of both attenuating changes in the rescued rLASV/IGR-CD. After generation of virus stocks, growth kinetics of both viruses were assessed by plaque assay (Fig. 1B–D) and quantitative reverse transcription PCR (RT-qPCR) (Fig. 1E, F) in Vero (IFN-deficient) and A549 (IFN-competent) cells at multiplicities of infection (MOIs) of 0.01 and 0.1 (Fig. 1B–F). Both viruses replicated to high titers in Vero cells. However, compared with rLASV-WT, rLASV/IGR-CD replicated at a slower rate and reached significantly lower peak titers in both Vero and A549 cells (Fig. 1B–F). A greater reduction of replication of rLASV/IGR-CD was seen in A549 compared with Vero cells. Plaques of rLASV/IGR-CD were smaller than those of rLASV-WT (Fig. 1D).

### Viral protein expression levels and virion morphology of rLASV/IGR-CD in vitro

**Fig. 2:**
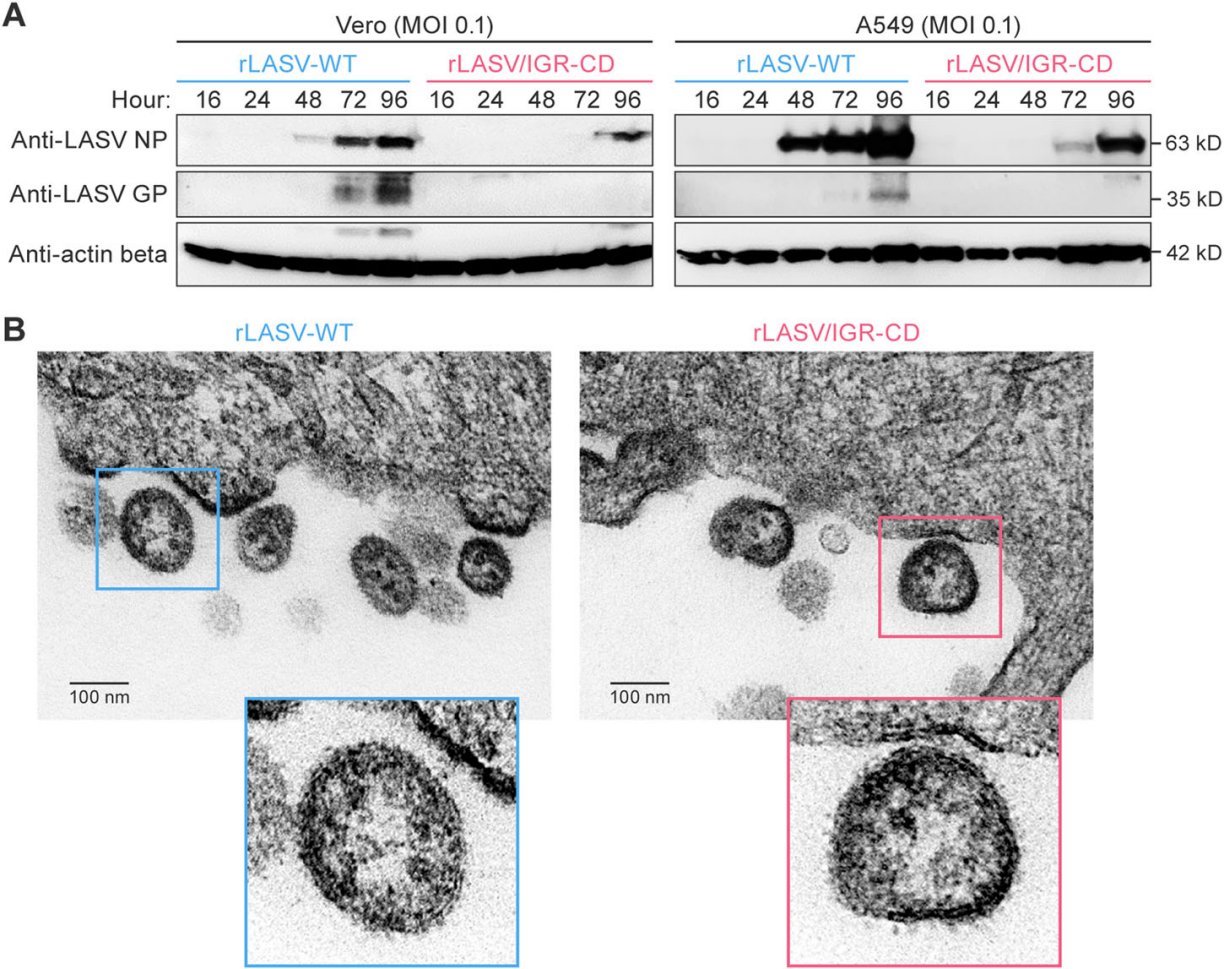
Viral protein expression levels and virion morphology of rLASV/IGR-CD in vitro. **A** Vero cells or A549 cells were exposed to rLASV-WT or rLASV/IGR-CD and cell lysates were collected at the indicated time points. Western blots were performed on cell lysates with antibodies to LASV NP, LASV GP, or actin beta (loading control). See Supplementary Fig. 4 for uncropped blots. **B** Resin blocks containing Vero cells infected with rLASV-WT or rLASV/IGR-CD were sectioned and scanned by a transmission electron microscope. Samples contained small quantities of LASV particles measuring 80–140 nm in diameter that were budding from the plasma membranes of infected cells and extracellular mature particles. Shown are two representative images of mature rLASV-WT and rLASV/IGR-CD virus particles in extracellular space of infected Vero cells. CD codon-deoptimized; GP glycoprotein; IGR intergenic region; LASV Lassa virus; MOI multiplicity of infection; NP nucleoprotein; rLASV recombinant LASV; WT wild-type.

We hypothesized that replicative fitness differences between rLASV-WT and rLASV/IGR-CD were at least in part due to differential viral protein expression. To evaluate this hypothesis, we prepared cell lysates from the previous growth kinetics experiments (MOI 0.1) and measured protein expression over time by western blot. Consistent with replication results (Fig. 1), NP expression was delayed in rLASV/IGR-CD-infected cells (first detection at 72–96 h post-exposure) compared with rLASV-WT-infected cells (48 h post-exposure), and GP was not detected in lysates from rLASV/IGR-CD-infected cells at any of the evaluated times, whereas GP was readily detected in lysates from rLASV-WT-infected cells at 72 h post-exposure (Fig. 2A). Additionally, cells infected with either rLASV-WT or rLASV/IGR-CD were collected and analyzed by electron microscopy (EM): no morphological differences between the two viruses (Fig. 2B) were observed, indicating that rLASV/IGR-CD replicative fitness loss was associated with impaired protein expression but not apparent structural abnormalities.

### Assessment of rLASV/IGR-CD stability during serial passages in cultured cells

Reversion to virulence is a major concern for LAV strategies. Therefore, we evaluated the genetic stability of rLASV/IGR-CD during three independent serial (18) passages in Vero cells, using an MOI of 0.01 and sampling cell culture supernatant (CCS) at 72 h post-infection. Plaque assays for these viruses require long incubation periods, making them impractical. Thus, we determined levels of viral RNA in CCS samples by RT-qPCR and converted them to equivalent plaque-forming units (ePFU). Virus genomes were sequenced after passages 13 and 18. rLASV/IGR-CD titers fluctuated (within 1 log_10_ up or down) but did not significantly change (Supplementary Fig. 2) over 18 serial passages, supporting the maintenance of replicative fitness in Vero cells, a cell line approved by the U.S. Food and Drug Administration for production of human vaccines. Compared with passage 1, the S and L segments acquired various non-synonymous mutations (Table 1 and Supplementary Table 1). In the S segment, change K143R in GPC and change R426G in NP were selected in the three replicates. In the L segment, change M1021I in L protein was selected in all three replicates and change T95K in Z was present in two out of three replicates.

**Table 1:** Assessment of rLASV/IGR-CD stability during serial passage in cultured Vero cells

Nucleotide position	Reference allele	Mutated allele	P13R1	P13R2	P13R3	P18R1	P18R2	P18R3	Amino-acid residue change	Affected protein*
**S segment**										
50	T	C	0	0	0	75.90	0	0	None (5′ UTR)	-
428	A	G	81.91	56.78	60.45	85.50	85.17	30.20	K143R	GPC
2125	T	C	0	98.16	0	96.49	100.00	87.59	R426G	NP
2740	T	C	0	62.06	0	0	99.56	32.63	T221A	NP
**L segment**										
812	G	A	0	0	0	0	0	28.26	A271T	L
1550	T	C	0	71.22	0	0	77.03	0	S517P	L
2100	C	T	0	41.92	0	0	64.68	0	A700V	L
3042	A	G	0	0	26.41	0	0	0	K1014R	L
3062	A	G	0	0	0	0	0	27.20	M1021V	L
3064	G	A	0	94.98	0	36.31	100.00	34.13	M1021I	L
4521	A	T	29.28	0	29.30	0	0	0	Y1507F	L
6837	C	G	0	0	0	54.19	0	0	None (IGR)	-
6862	CG^a^	C	0	0	0	0	28.91	0	None (IGR)	-
6964	G	T	0	52.41	0	0	54.26	27.03	T95K	Z

rLASV/IGR-CD acquires multiple mutations in late passages in vitro

Table showing the proportion (percent of total reads) of non-synonymous mutations in the samples. A value of 100 indicates that all reads had the mutated allele at the specified position, and 0 indicates that no mutated alleles were seen at the indicated position in those samples.

*GPC glycoprotein precursor, *IGR* intergenic region, *L* large protein, *NP* nucleoprotein, *P* passage, *R* replicate, *UTR* untranslated region, *Z* zinc-binding protein.

^a^CG to C mutation at position 6862 (L segment) indicates a single nucleotide deletion.

### rLASV/IGR-CD is attenuated in vivo

**Fig. 3:**
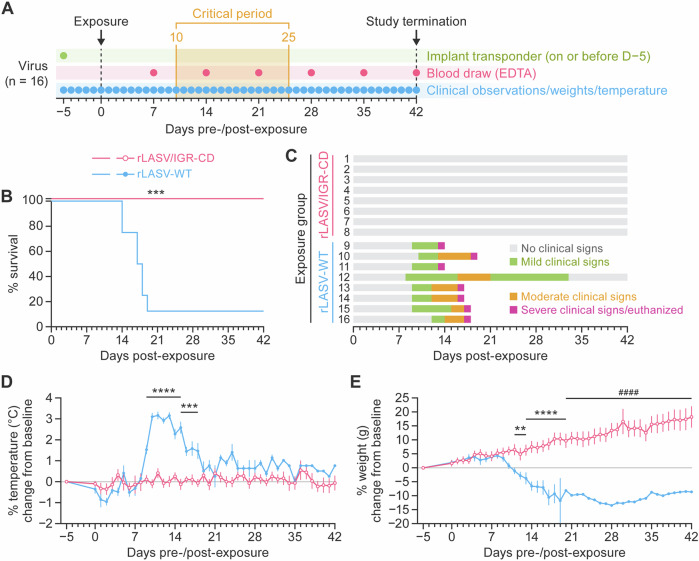
rLASV/IGR-CD is attenuated in vivo. **A** Study design, **B** Kaplan–Meier survival curve, and **C**–**E** clinical observations of strain 13 guinea pigs exposed to 10^5^ PFU of rLASV-WT (*n* = 8) or rLASV/IGR-CD (*n* = 8). “Mild signs” indicates ruffled fur, increased respiration rate, and slightly reduced activity. “Moderate signs” indicates ruffled fur, partially closed eyes, increased respiration rate with abdominal activity, and significantly reduced activity. “Severe signs” indicates respiratory distress, closed or mostly closed eyes, severe lethargy, and cyanosis. Error bars indicate standard error of the mean. **p < 0.005, ***p < 0.0005, ****p < 0.0001. CD codon-deo*p*timized; EDTA ethylenediaminetetraacetic acid; IGR intergenic region; LASV Lassa virus; rLASV recombinant LASV; PFU plaque-forming units; WT wild-type.

To evaluate the virulence of rLASV/IGR-CD, we exposed strain 13 guinea pigs, an established lethal model of LF^46^, subcutaneously to 10^5^ PFU of rLASV-WT (*n* = 8) or rLASV/IGR-CD (*n* = 8) and evaluated the animals by daily observation and weekly blood sampling until study termination on Day 42 post-exposure (Fig. 3A). Consistent with previous findings^46^, most animals (seven of eight) exposed to rLASV-WT met euthanasia criteria by Day 14–19 (Fig. 3B) and all animals developed clinical signs (e.g., ruffled coat, increased respiratory rate, elevated temperatures, weight loss) (Fig. 3C–E). In stark contrast, all rLASV/IGR-CD-exposed animals survived to study termination (Fig. 3B) without developing clinical signs of disease (Fig. 3C, D), and consistently gained weight throughout the study (Fig. 3E).

### Tissue viral RNA levels, virus-specific immunoglobulin G titers, and cytokine gene expression in guinea pigs support in vivo attenuation of rLASV/IGR-CD

**Fig. 4:**
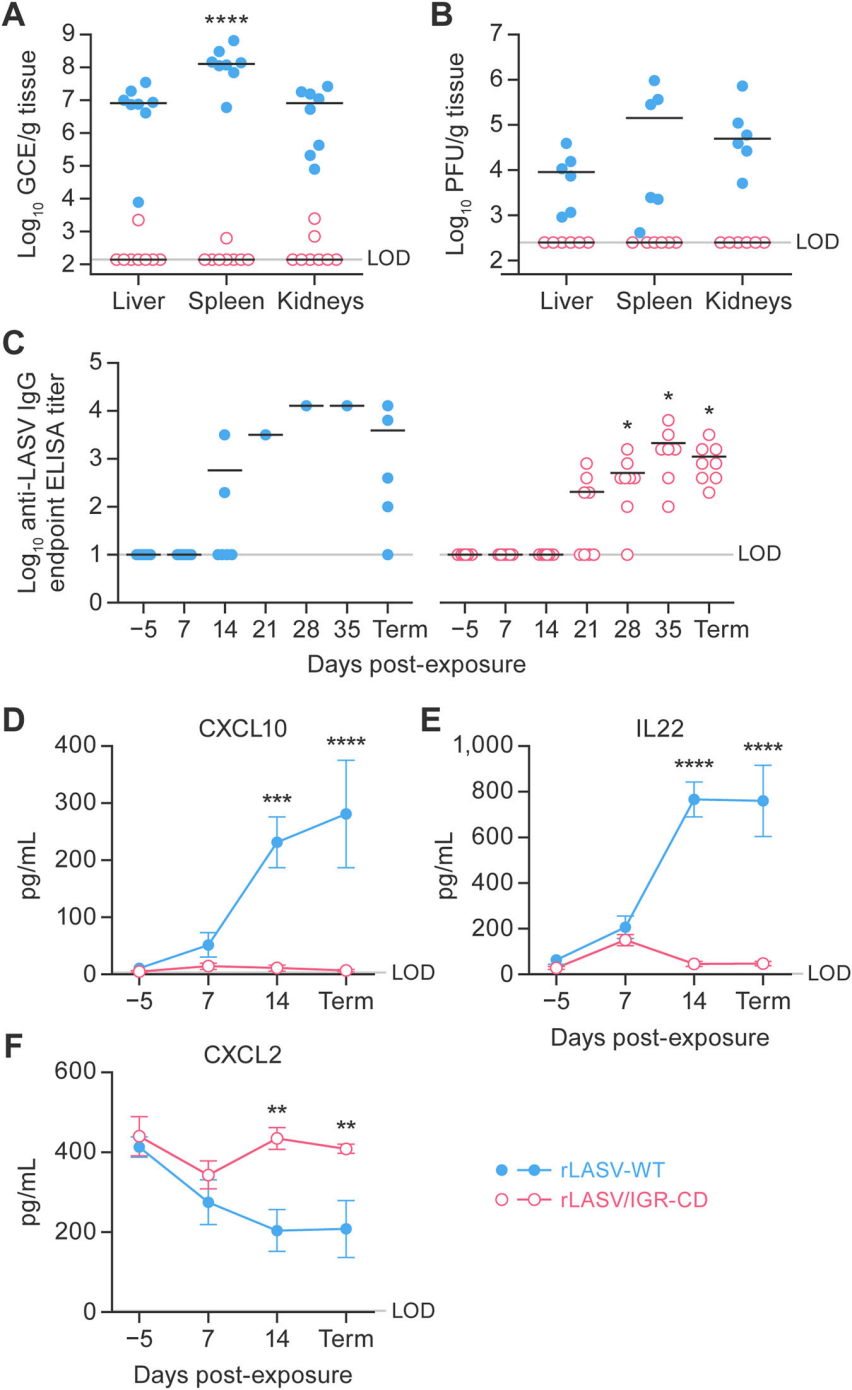
Tissue viral RNA levels, LASV antigen-specific IgG titers, and cytokine gene expression in guinea pigs support in vivo attenuation of rLASV/IGR-CD. **A** RT-qPCR and **B** plaque assays were performed on homogenates from livers, spleens, and kidneys collected at necropsy to determine virus titers, and **C** anti-LASV IgG ELISA and multiplex cytokine panel analysis containing analytes **D** CXCL10, **E** IL22, and **F** CXCL2 were performed using plasma collected at the indicated time points during the study depicted in Fig. 3. Error bars indicate standard error of the mean. *p < 0.05, **p < 0.005, ***p < 0.0005, ****p < 0.0001. CXCL C-X-C motif chemokine ligand; ELISA enzyme-linked immunosorbent assay; GCE genome copy equivalents; Ig, immunoglobulin; IGR intergenic region; IL, interleukin; LASV Lassa virus; LOD limit of detection; PFU plaque-forming units; rLASV recombinant LASV; Term, Day 42, the terminal day of the study, i.e., time point of euthanasia; WT wild-type.

Viral RNA and infectious virus were detected at necropsy in tissues from all animals exposed to rLASV-WT; however, only a small amount of viral RNA could be detected in the liver and spleen (of one animal) and in the kidneys (of two animals) exposed to rLASV/IGR-CD and infectious virus was not detected in all tissues examined (Fig. 4A, B).

Two of six guinea pigs remaining at Day 14 after exposure to rLASV-WT had detectable levels of anti-LASV immunglobulin G (IgG). Only one rLASV-WT-exposed animal survived past Day 14, and anti-LASV IgG was detected at high levels in plasma of this animal throughout the remainder of the study. In rLASV/IGR-CD-exposed animals, anti-LASV IgG was first detected at Day 21, was detected at high levels in all animals by Day 35 and was maintained through Day 42 (Fig. 4C).

Innate immunity was analyzed via a 16-plex cytokine assay. Compared with pre-exposure baselines, significant elevations in C-X-C motif chemokine ligand 10 (CXCL10, formerly IP-10) and interleukin 22 (IL22) concentrations were measured in rLASV-WT-exposed animals at Day 14 and at necropsy (Day 42); no notable change in these cytokine concentrations occurred at any time point over the course of the study in rLASV/IGR-CD-exposed animals. Compared with baselines, CXCL2 (formerly MIP-2) concentrations were significantly decreased at Day 14 and at necropsy in rLASV-WT-exposed animals; comparatively, no appreciable change was measured over similar time points in rLASV/IGR-CD-exposed animals (Fig. 4D–F). Between-group analysis showed no significant differences in the concentrations of other measured analytes.

### Histopathology of liver and lung tissues supports in vivo attenuation of rLASV/IGR-CD

**Fig. 5:**
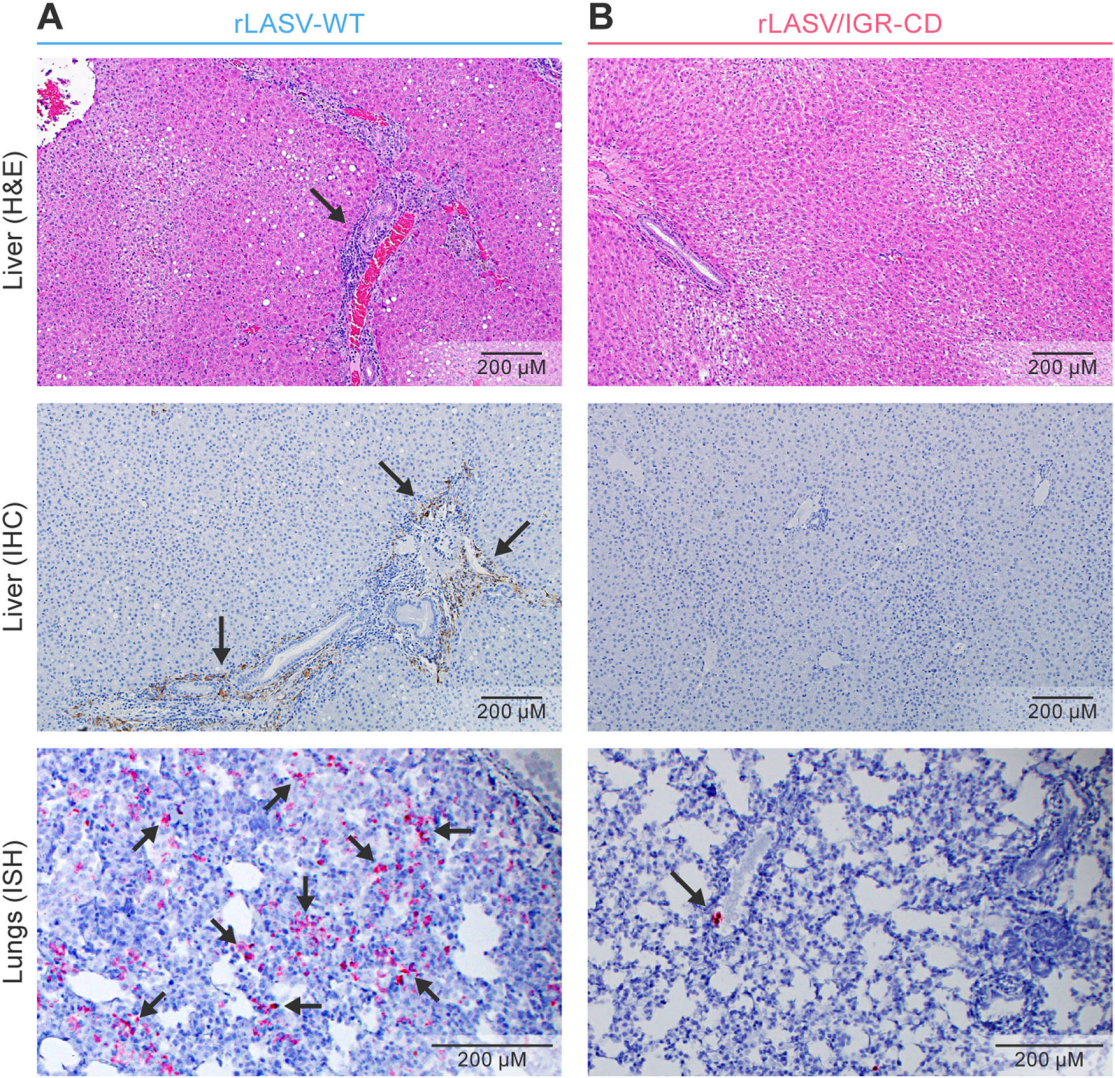
Histopathology of liver and lung tissues support in vivo attenuation of rLASV/IGR-CD. Representative images of H&E and IHC staining of liver tissues and ISH staining of lung tissues taken at necropsy during the study depicted in Fig. 3. Black arrows depict liver degradation and single-cell necrosis (**A**, top), viral antigen (**A**, middle), and viral RNA in a single cell (**A**, bottom; **B**, bottom). CD codon-deoptimized; H&E hematoxylin and eosin; IHC immunohistochemistry; ISH in situ hybridization; LASV Lassa virus; rLASV recombinant LASV; WT wild-type.

Histopathological examination of rLASV-WT-exposed animals revealed typical findings of LASV-induced disease, including hepatocellular degeneration with some individual single-cell necrosis, and viral antigen (immunohistochemistry [IHC]) and RNA (in situ hybridization [ISH]) was detectable in abundance in liver and lungs (Fig. 5A). In the single surviving rLASV-WT-exposed animal, significant pathologic findings and viral antigen and RNA were not detected. Histopathological abnormalities were not observed in the tissues of rLASV/IGR-CD-exposed guinea pigs and viral antigen could not be detected. Likewise, viral RNA was not detected by ISH in tissues of rLASV/IGR-CD-exposed animals, except in a cluster of a few cells in the lungs of one guinea pig (Fig. 5B). Overall, these results demonstrated that rLASV/IGR-CD was highly attenuated in vivo.

### rLASV/IGR-CD-vaccinated strain 13 and Hartley guinea pigs are protected against LASV-induced disease

**Fig. 6:**
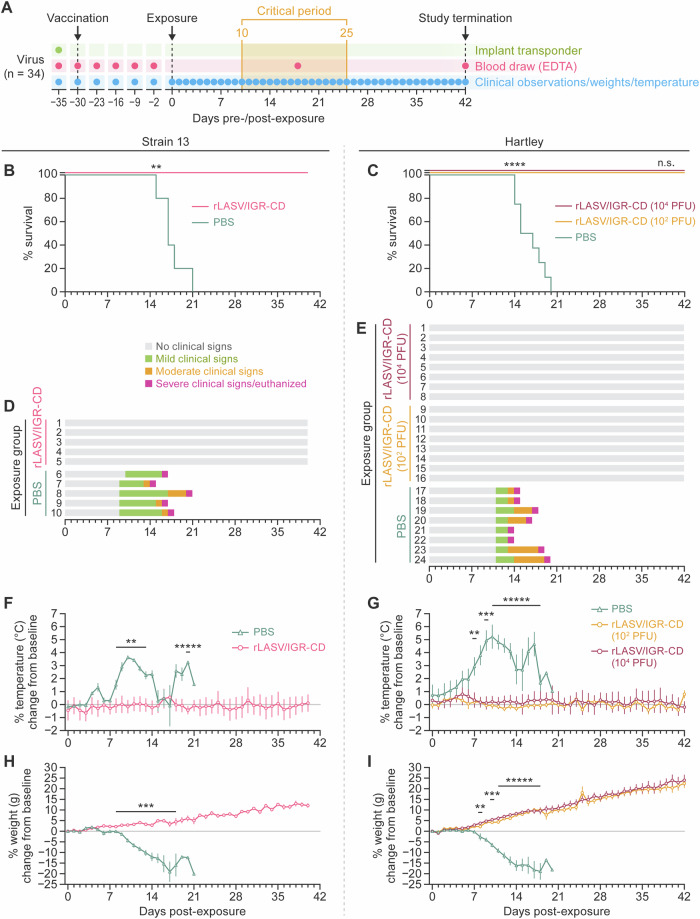
rLASV/IGR-CD-vaccinated strain 13 and Hartley guinea pigs are protected against LASV-induced disease*.* **A** Study design, **B**–**C** Kaplan–Meier survival curves, and **C**–**I** clinical observations of strain 13 (*n* = 5) and Hartley (*n* = 8) guinea pigs vaccinated with PBS (mock vaccine) or 10^5^ PFU (strain 13) or 10^2^ PFU or 10^4^ PFU (Hartley) of rLASV/IGR-CD and exposed to 10^5^ PFU of rLASV-WT (strain 13) or 10^4^ PFU of GPA-LASV (Hartley). “Mild signs” indicates ruffled fur, increased respiration rate, and slightly reduced activity. “Moderate signs” indicates ruffled fur, partially closed eyes, increased respiration rate with abdominal activity, and significantly reduced activity. “Severe signs” indicates respiratory distress, closed or mostly closed eyes, severe lethargy, and cyanosis. Error bars indicate standard error of the mean. **p < 0.005, ***p < 0.0005, ****p < 0.00005. CD, codon-deoptimized; EDTA, ethylenediaminetetraacetic acid; GPA-LASV, guinea pig-adapted LASV; IGR, intergenic region; LASV, Lassa virus; rLASV, recombinant LASV; PBS, phosphate-buffered saline; PFU, plaque-forming units; WT, wild-type.

To evaluate whether rLASV/IGR-CD inoculation results in protection against a typically lethal LASV exposure, strain 13 (*n* = 5) and Hartley (*n* = 8) guinea pigs were subcutaneously inoculated with a mock vaccine (phosphate-buffered saline [PBS]) or 10^5^ PFU (strain 13) or 10^2^ PFU or 10^4^ PFU (Hartley) of rLASV/IGR-CD and evaluated daily for clinical signs of disease (including changes in weight and temperature) and weekly via blood sampling for 30 d after inoculation. Animals were subsequently exposed subcutaneously to 10^5^ PFU of rLASV-WT (strain 13) or intraperitoneally to 10^4^ PFU of GPA-LASV (Hartley). Animals were monitored daily for clinical signs of disease (including changes in weight and temperature) and blood was sampled at Day 16 and at study termination on Day 42 post-exposure (Fig. 6A). As expected, all mock-vaccinated (PBS) animals developed several clinical signs (e.g., ruffled coat, increased respiratory rate, elevated temperatures, weight loss) and met euthanasia criteria within 21 d post-exposure (Fig. 6B–I). In stark contrast, all rLASV/IGR-CD-vaccinated animals survived to study termination (Fig. 6B, C) without developing clinical signs of disease (Fig. 6D–G) and consistently gained weight (Fig. 6H, I)

### Viral RNA levels and histopathological findings in guinea pigs vaccinated with rLASV/IGR-CD and exposed to rLASV-WT

**Fig. 7:**
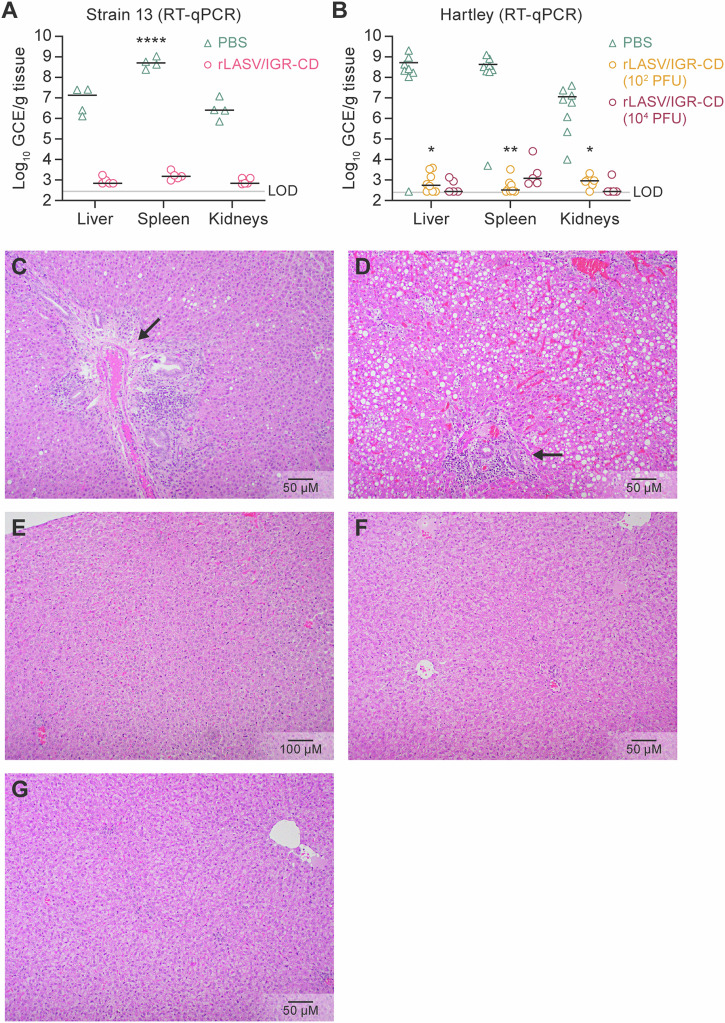
Viral RNA levels and histopathological findings in guinea pigs vaccinated with rLASV/IGR-CD and exposed to rLASV-WT. RT-qPCR assays were performed on homogenates from livers, spleens, and kidneys collected at necropsy to determine virus titers during the study depicted in Fig. 6 in **A** strain 13 and **B** Hartley guinea pigs. Representative images of H&E staining of liver issues taken at necropsy during the study depicted in Fig. 6, in which animals were vaccinated with **C** PBS (strain 13), **D** PBS (Hartley), **E** 10^5^ PFU rLASV/IGR-CD (strain 13), **F** 10^2^ PFU rLASV/IGR-CD, or **G** 10^4^ PFU rLASV/IGR-CD. Arrows depict liver degradation and single-cell necrosis. *p < 0.05, **p < 0.005, ***p < 0.0005, ****p < 0.00005. CD codon-deoptimized; GCE genome copy equivalents; IGR intergenic region; LASV; Lassa virus; LOD limit of detection; rLASV recombinant LASV; PBS phosphate-buffered saline; PFU plaque-forming units; RT-qPCR real-time reverse transcription polymerase chain reaction; WT wild-type.

High virus titers were found in all mock-vaccinated and rLASV-WT- exposed animals (Fig. 7A, B), and all animals had significant histopathological lesions consistent with LASV infection, including hepatocellular degeneration and single-cell necrosis (Fig. 7C, D). In contrast, virus titers were low or undetectable in all rLASV/IGR-CD-vaccinated animals (Fig. 7A, B), and no significant histopathologic lesions were observed (Fig. 7E–G).

### Evaluation of rLASV/IGR-CD-induced immunity in strain 13 guinea pigs

**Fig. 8:**
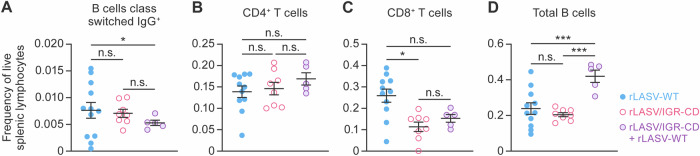
Evaluation of rLASV/IGR-CD induced immunity in strain 13 guinea pigs. Cells isolated from spleens were stained with a newly developed flow cytometry panel (gating strategy detailed in Supplemental Fig. 3) and run on a flow cytometer to identify distinct populations of splenic immune cells. Frequencies of live splenic lymphocytes were examined across the groups for **A** class-switched IgG^+^ B cells, and total populations of **B** CD4^+^ T cells, **C** CD8^+^ T cells, and **D** total B cells. Error bars indicate standard error of the mean. n.s.= *p* < 0.05 *p < 0.05, ***p < 0.0005. CD codon deo*p*timized; IgG immunoglobulin G; IGR intergenic region; n.s. not significant; rLAS recombinant Lassa virus; WT wild-type.

To gain insight into correlates of rLASV/IGR-CD-mediated protection, we characterized the underlying cellular immune responses in strain 13 guinea pigs. Given the limited availability of relevant guinea-pig-specific reagents and assays, we developed a nine-color flow cytometric assay to immunophenotype peripheral and tissue-associated guinea pig immune cells (Supplementary Fig. 3). Splenocytes isolated from animals exposed to rLASV-WT and rLASV/IGR-CD had similar B and CD4^+^ T cell frequencies (Fig. 8A, B); a lower frequency of CD8^+^ T cells was detected in LASV/IGR-CD-exposed as compared with rLASV-WT-exposed animals (Fig. 8C). Compared with animals exposed to rLASV/IGR-CD alone, animals vaccinated with rLASV/IGR-CD and subsequently exposed to rLASV-WT exhibited increased, but not statistically significantly, frequencies of CD8^+^ T cells (Fig. 8C). A striking increase in the frequency of total splenic B cells (Fig. 8D) was noted in vaccinated/exposed animals relative to groups with either exposure or vaccination alone. Overall, these results demonstrated that rLASV/IGR-CD vaccination is associated with a reduction in the frequency of CD8^+^ T cells and protective vaccination-induced immune responses are associated with increased numbers of B cells in the convalescent spleen. Furthermore, these results showed that rLASV/IGR-CD vaccination protects not only from fatal outcome but also from clinical disease and pathology after exposure to rLASV-WT.

## Discussion

Vaccines are important tools for preventing infectious diseases and improving One Health. Prior to use in a clinical setting, vaccine candidates require extensive evaluation of safety, stability, and preventive efficacy against infection and/or disease. The development of a safe, effective vaccine to prevent or improve outcomes in LF is a global priority^3,35^, and proposed candidates include recombinant viral vector vaccines based on the mammarenavirus Mopeia virus^47^, the orthopoxvirus vaccinia virus^48,49^, the vesiculovirus vesicular stomatitis Indiana virus^50–52^, and the orthoflavivirus yellow fever virus^53,54^. Although viral vector vaccines may provide long-lasting protection, the uncertain impact of pre-existing, or subsequent, vector-targeted immune responses on vaccine effectiveness remains a significant and non-theoretical concern. For example, in individuals vaccinated with Ad5-nCoV, an adenoviral vector vaccine against SARS-CoV-2, high levels of pre-existing Ad5-targeted antibodies were associated with significantly lower humoral and cell-mediated immune responses to SARS-CoV-2 after vaccination^55^. Vector-targeted immunity can also led to harm, i.e., an increased risk of infection, as seen in Ad5-based HIV/AIDS vaccine studies^56^. Furthermore, viral vector vaccines may be associated with significant and severe side effects, as seen in patients with immune thrombocytopenia associated with life-threatening thrombosis after vaccination with the ChAdOx1 adenovirus-based SARS-CoV-2 vaccine^57,58^. Accordingly, uncertainty around immunogenicity (including a plausible impact of vector-targeted immunity) and safety are critical issues being assessed in phase 1/2 clinical trials of the most advanced LASV vaccine candidate, rVSV∆G-LASV-GPC^59^.

The only currently approved mammarenavirus vaccine for humans is the live-attenuated Junín virus Candid#1, which is approved for use in Argentina and has investigational new drug status in the U.S^60–63^. LAVs frequently trigger lasting robust cell-mediated and humoral immune responses^64^. However, concerns about reversion to virulence events (through mutation, recombination, or reassortment with wild-type strains) obligate careful preclinical evaluation of this risk, that, at minimum, establishes an extremely low likelihood of reversion^65^. Natural genetic inter-species reassortment appears to be frequent among certain arenavirids that infect snakes (reptarenaviruses) but rare among mammarenaviruses^66–68^. Reassortment has been shown experimentally for several African mammarenaviruses, including LASV and Mopeia virus^69–72^, and recent large-scale sequencing efforts support the occurrence, albeit at low frequency, of natural LASV reassortment: three out of 194 characterized LASV isolates are considered putative reassortants^73^.

Our previous work^43,44^ characterized two LASV LAV candidates, rLASV/IGR(S-S) and rLASV-GPC/CD, each containing a genetic determinant of attenuation in the L segment and the S segment, respectively. As for LCMV counterparts rLCMV/IGR(S-S)^74^ and rLCMV-GPC/CD, we demonstrated that rLASV/IGR(S-S) and rLASV-GPC/CD^75^ have excellent safety profiles as LAV candidates. Attenuation of rLASV/IGR(S-S) and rLASV-GPC/CD is not based on specific mutations in the *L* or *GP* genes resulting in amino acid residue changes, but rather on two distinct features of their genome organization and associated gene expression profile: 1) Stably decreased expression of the GP precursor GPC still enables production of progeny virus in Vero cells (a cell substrate approved by the U.S. Food and Drug Administration for production of human vaccines), compatible with the levels required for vaccine production; and 2) Replacement of the L-IGR by the S-IGR, resulting in sustained altered expression of L protein and Z. However, coinfection of laboratory mice with rLCMV/IGR(S-S) and rLCMV-GPC/CD resulted in the rapid generation of WT-like reassortants that caused lymphocytic choriomeningitis with fatal outcome^45^. This precedent finding raised the concern that a virulent LASV reassortant could emerge in a person immunized with rLASV-GPC/CD and infected (around the same time) with a naturally circulating LASV strain attenuated via genetic determinants present only in the L segment. The same concern applies to immunization with rLASV/IGR(S-S) and exposure to a circulating LASV strain attenuated via genetic determinants present only in the S segment. Although the probability of these reversion events is highly unlikely, safety risk must nonetheless be addressed. Our previous results with rLCMV/IGR-CD^45^ provided a strong rationale for the development of the novel rLASV/IGR-CD generated and characterized in the present work, and addressed this safety concern.

Compared with rLASV-WT, inoculation with rLASV/IGR-CD resulted in decreased infectious virus titers and viral RNA at all evaluated time points after infection in both A549 and Vero cells. Moreover, the smaller plaque size of rLASV/IGR-CD further supported its attenuated phenotype. We also demonstrated a reduction in viral protein expression levels in rLASV/IGR-CD-infected cells. Despite production of high ( ≈ 10^6^ PFU/mL) peak titers of infectious virus (Fig. 1B), we did not detect GP by western blot in Vero cells infected with rLASV/IGR-CD (Fig. 2A). We did not quantify spike density on the surface of rLASV/IGR-CD particles produced in Vero cells; however, visual inspection of the transmission electron microscopy images suggests that LASV and rLASV/IGR-CD particles have a similar spike density (Fig. 2B).

Importantly, rLASV/IGR-CD was both highly attenuated and immunogenic in strain 13 guinea pigs, in which it triggered robust anti-LASV IgG responses and generated splenocyte CD4^+^ T cell population frequencies similar to those detected after rLASV-WT-exposure. An important limitation of our immunoprofiling after rLASV/IGR-CD vaccination was the lack of quantification of LASV-specific T cell responses. To this aim we developed an intracellular cytokine staining assay (ICS) to detect IFNG production by guinea pig CD8^+^ T cells. We found a robust production of IFNG by CD8^+^ T cells from guinea pig peripheral blood mononuclear cells (PBMCs) upon exposure to PMA/Ionomycin for 6 h, with the final 5 h of activation taking place in the presence of brefeldin A and monensin. Our only source of guinea pig cells potentially containing LASV antigen-specific T cells were the splenocytes banked during our studies. We used rLASV/IGR-CD and mock-infected splenocytes to stimulate freshly thawed splenocytes for 18 h. Across multiple animals tested, we did not observe differences in levels of IFNG produced by the two populations (data not shown). Future studies, beyond the scope of the present work, will address whether LASV-peptide pools can result in activation of virus-specific CD8^+^ T cells.

Guinea pigs immunized with rLASV/IGR-CD did not exhibit clinical signs or viremia at any observation time over the duration of the study (42 d). At the experimental endpoint of the study, LASV RNA was detected by RT-qPCR at very low levels in one liver, one spleen, and two kidney samples out of 24 samples examined for the presence of viral RNA in guinea pigs immunized with rLASV/IGR-CD. However, infectious virus was not detected in any of the 24 tissue samples examined from guinea pigs immunized with rLASV/IGR-CD. Nevertheless, additional studies will be needed to unequivocally determine whether in rare occasions rLASV/IGR CD might be able to persist for some time.

As compared with rLASV-WT exposure, we noted distinct differences in several key cytokine responses after inoculation with rLASV/IGR-CD. Increased levels of CXCL10 decrease T cell proliferation and lead to lymphopenia, which can lead to more severe viral disease^76^. Increased CXCL10 is also associated with impaired restoration of the endothelium after disruption, which may play a role in the hemorrhagic manifestations of LF^77^. Similarly, IL22 is associated with pro-inflammatory signaling^78–80^ and blood–brain barrier breakdown and lymphocyte infiltration^81^. Macrophage-derived CXCL2 contributes to several inflammatory diseases, such as glomerulonephritis and meningitis^82^. Notably, levels of CXCL10, IL22, and CXCL2 were essentially unchanged or detected at low levels in guinea pigs inoculated with rLASV/IGR-CD, supporting the notion that rLASV/IGR-CD does not promote inflammation-related pathology seen after rLASV-WT exposure. These encouraging data led us to determine the protective efficacy of rLASV/IGR-CD in two lethal guinea pig models of LF. Inbred strain 13 guinea pigs and outbred Hartley guinea pigs were fully protected from a typically lethal LASV exposure. Furthermore, none of the rLASV/IGR-CD-inoculated animals developed any signs of clinical illness, consistently gained weight, and remained afebrile at all evaluated time points after lethal LASV exposure. Consistent with the clinical readout, no pathological abnormality was found in organs of rLASV/IGR-CD-vaccinated animals after lethal LASV exposure.

Genetic stability is a critical feature of any LAV to prevent reversion to a virulent form. We used RT-qPCR to determine titers of rLASV/IGR-CD in CCS during serial passage in Vero cells. This approach does not assess the possible contribution of defective viral genomes to reduced virus infectivity. However, the absence of significant change in the estimated titers of rLASV/IGR-CD in CCS even after prolonged serial low-MOI passage suggests that defective viral genomes were likely not present in CCS at levels that impacted experimental outcomes. Moreover, rLASV/IGR-CD was genetically stable during serial passage in Vero cells, and, after 18 passages, only three mutations (GPC [K143R], NP [R426G], and L [M1021I]) were selected in all three replicates and an additional mutation (Z [T95K]) was selected in two replicates. It should be noted that the observed mutations could be due to adaptations to Vero cells rather than attenuation-driven compensation. However, rLASV/IGR-CD titers did not increase after acquisition of the identified mutations. Future studies will be required to elucidate the impact of these mutations on replication and attenuation. Regardless, our data indicate that reversion of rLASV/IGR-CD to a form with increased virulence is highly unlikely.

Our previous findings with LCMV^45,74,75^ and LASV^43,44^, together with the data shown here, indicate that the incorporation of genetic determinants of attenuation in both the S segment (a codon-deoptimized *GP* gene) and L segment (replacement of the L segment IGR with the S segment IGR) is an attractive approach to develop mammarenavirus LAV candidates with both enhanced safety and desirable immunogenicity afforded by maintenance of an antigenic profile similar to the original pathogenic strain. It should be noted that demand for an LF vaccine may reach 100 million doses, including from Nigeria, where LASV lineages I–III are circulating^83^. We designed and evaluated our candidate LAV in the background of LASV isolate Josiah, a lineage IV LASV that circulates in Guinea, Liberia, and Sierra Leone. Immunization with the LF LAV candidate reassortant ML29 expressing NP and GP antigens of isolate Josiah protects guinea pigs against a typically lethal exposure to homotypic virus and the distantly related LASV isolate 803213 (lineage II from south-central Nigeria). We therefore predict that rLASV/IGR-CD will induce protective immunity against lineages II and IV LASV. Based on the close relationships of lineages II and III, we also expect that rLASV/IGR-CD will provide protection against lineage III LASV (north-central Nigeria). We cannot exclude that rLASV/IGR-CD may provide poor cross-immunogenicity and protection against the most distantly related LASV isolate LP (lineage I). However, a vesicular stomatitis Indiana virus-based LF vaccine using the LASV isolate Josiah GP as the immunogen conferred protection against LASV isolate Pinneo (lineage I), suggesting that rLASV/IGR-CD could also protect against lineage I LASV^51^.

Our results with rLCMV/IGR-CD^45^ support the hypothesis that the combination of safety features we incorporated into rLASV/IGR-CD will mitigate against an unlikely but very consequential scenario, i.e., the generation of reassortant viruses with increased virulence enabled by unnoticed exposure to naturally circulating LASV variants around the time of vaccination with rLASV/IGR-CD. It should be noted that a single low dose (10^2^ PFU) of rLASV/IGR-CD was sufficient to provide complete protection against a typically lethal dose of LASV; in contrast, the protective dose (10^6^ PFU) of the rVSVΔGP-LASV-GPC candidate was 10,000x higher in the same guinea pig model^84^. Vector-independence, resistance to reversion, and this dosing advantage supports further preclinical development, including cost-effective production, of rLASV/IGR-CD as a vaccine candidate against LF. Indeed, the prioritized global urgency to identify safe and effective vaccines for LF argues for next-step investigation in a nonhuman primate model of LF to determine the lowest single dose of rLASV/IGR-CD that can provide short and long-term immunogenicity and protective efficacy.

## Materials and methods

### Cell lines

The sources and growing conditions of human adenocarcinoma alveolar basal epithelial A549, human embryonic kidney epithelial HEK293T/17, and grivet (*Chlorocebus aethiops* (Linnaeus, 1758)) kidney epithelial Vero (CCL-81) and Vero E6 (BEI Resources, Manassas, VA, USA; #BR596) cells were described previously^43,44^.

### Viruses

All experiments associated with replicating viruses were performed under maximum (biosafety level 4 [BSL-4]) containment at the Integrated Research Facility at Fort Detrick (IRF-Frederick; Fort Detrick, Frederick, MD, USA), according to approved standard operating procedures. LASV isolate Josiah^85^ (LASV) and domesticated guinea-pig-adapted LASV (GPA-LASV) were provided by the U.S. Army Medical Research Institute of Infectious Diseases (USAMRIID; Fort Detrick, Frederick, MD, USA). LASV, recombinant LASV (rLASV-WT), and rLASV/IGR-CD were grown, harvested, and titered by plaque assay as described previously^43,44^.

### Plasmids

pCAGGS expression plasmids encoding L protein (pCAGGS LASV-L), and nucleoprotein (pCAGGS LASV-NP) of LASV, and Escherichia phage T7 RNA polymerase (pCAGGS-T7) have been described^43,44^. Plasmids encoding the LASV (isolate Josiah) small (S) and large (L) segment antigenomes (ag) under control of the T7 promoter (pT7-LASV-S(ag))—pT7-LASV-L(ag), pT7-LASV-L-IGR/S-S(ag), and pT7-LASV-GPC/CD(ag))—were generated as described^43,44^.

### Rescue and propagation of rLASVs

HEK293T/17 cells (7 × 10^5^ cells per well, 6-well plate format) were cotransfected with pCAGGS LASV-NP (0.8 µg), pCAGGS LASV-L (1.0 µg), pCAGGS-T7 (1.0 µg), and

- pT7-LASV-S(ag) (0.8 µg) + pT7-LASV-L(ag) (1.4 µg) to create rLASV-WT or
- pT7-LASV-GPC/CD(ag) (0.8 µg) + pT7-LASV-L-IGR/S-S(ag) (1.4 µg) to create rLASV/IGR-CD

using 2.0 µL/µg DNA of Lipofectamine 2000 (Thermo Fisher Scientific, Waltham, MA, USA) according to the manufacturer’s instructions. At 6 h post-transfection, mixtures were replaced with Dulbecco’s Modified Eagle’s Medium (DMEM; Thermo Fisher Scientific) containing 2% heat-inactivated fetal bovine serum (FBS; MilliporeSigma, Burlington, MA, USA). Tissue-culture supernatants were collected on Day 3 and Day 6 post-transfection. On Day 6, transfected HEK293T/T17 cells were cocultured (1:1) with Vero cells. Tissue-culture supernatants were collected 4, 7, 11, and 15 d later. Virus titers were determined by plaque assay in Vero E6 cells, as described previously^43,44^. Plaques were counted manually on Day 6.

### Virus growth kinetics

Vero (CCL-81) and A549 cells were seeded in 96-well plates (1 × 10^4^ cells per well) and exposed to rLASV-WT or rLASV/IGR-CD, at multiplicities of infection (MOIs) of 0.01 and 0.1. Tissue-culture supernatants were collected at 4, 8, 16, 24, 48, 72, and 96 h post-exposure. Virus growth kinetics comparisons were performed via plaque assay and RT-qPCR, as described previously^43,44^.

### Western blot analysis

Vero and A549 cells were exposed to rLASV-WT or rLASV/IGR-CD (MOIs of 0.01 and 0.1). At 16, 24, 48, 72, and 96 h post-exposure, cells were lysed with NuPAGE LDS Sample Buffer (4X) (Thermo Fisher Scientific) before removing them from the BSL-4 laboratory. Cell lysates were resolved into 4‒12% Bis-Tris NuPAGE gels (Thermo Fisher Scientific) and transferred to nitrocellulose membranes. Membranes were blocked with 5% nonfat milk in phosphate-buffered saline (PBS) (Thermo Fisher Scientific) with 0.1% TWEEN (MilliporeSigma) for 1 h at room temperature. Membranes were incubated overnight with anti-LASV GP2 polyclonal antibody, anti-LASV NP monoclonal antibody, or an anti-actin beta antibody as loading control, followed by incubation with horseradish-peroxidase-conjugated secondary antibodies (MilliporeSigma), all described previously^43,44^.

### Electron microscopy

Vero cells were infected with rLASV-WT or rLASV/IGR-CD at an MOI of 5.0 and incubated in DMEM containing 2% heat-inactivated FBS for 72 h. Following incubation, the tissue-culture media were removed, and cells were washed in PBS. For conventional transmission electron microscopy, cells were preserved in 2.5% Glutaraldehyde (E.M. Sciences, Warrington, PA, USA), in Millonig’s Sodium Phosphate Buffer (Tousimis Research, Rockville, MD, USA). After fixation, the cells were washed in Millonig’s Buffer, incubated in 1.0% osmium tetroxide (E.M. Sciences), en bloc stained with uranyl acetate (E.M. Sciences), dehydrated in a series of graded ethanols, and infiltrated and embedded in Spurr Resin (E.M. Sciences). Resin blocks containing the cells were sliced to ultra-thin thickness, and 70-nm sections were collected on 150 mesh copper grids, stained with lead citrate, and examined in an FEI Tecnai Transmission Electron Microscope, operating at 80 kV. Both the rLASV-WT and rLASV/IGR-CD samples contained large numbers of cells and over 2,000 individual cell sections were examined from each sample.

### Genetic stability assessment

The genetic stability of rLASV/IGR-CD was evaluated in cell culture after being serially passaged 20 times in Vero cells. Briefly, Vero cells were exposed to rLASV/IGR-CD in a 6-well plate (passage 1) at an MOI of 0.01. At 72 h post-exposure, tissue-culture supernatants (passage 2) were collected, and virus titers were measured by RT-qPCR. Then, fresh Vero cells were exposed to passage 2 supernatants (MOI = 0.01) to generate passage 3. This process was repeated to generate 18 serial passages.

Given the challenges associated with detection of infectious LASV by classic plaque assays, genomic copy equivalents (GCEs) were assayed by RT-qPCR. ePFU during serial passages of rLASV/IGR-CD in Vero cells was derived by using the same serial dilutions of the rLASV/IGR-CD stock to determine infectious titer by plaque assay and levels of viral genome RNA by RT-qPCR. For each dilution, the ratio of GCE to PFU values was calculated by dividing GCEs by PFU values. This information was used to assess ePFU values during serial passages of rLASV/IGR-CD in Vero cells to identify the time point with the most similar GCE:PFU ratio, which informed the incubation period of 72 h between passages. Conversion to ePFU was based on the value derived from the ratio of GCEs per mL to PFU/mL at 72 h post-infection.

Passages 2, 13, and 18 were inactivated with TRIzol LS (Thermo Fisher Scientific), and viral RNA was extracted using the KingFisher Sample Purification System and MagMAX Viral Pathogen Nucleic Acid Isolation Kit. Briefly, 400 µL of inactivated sample was added to 550 µL of binding solution and magnetic beads. After binding and washing steps (with 80% ethanol [VWR, Radnor, PA, USA]), samples were eluted into 50 µL of elution buffer (Thermo Fisher Scientific). Purified RNA was converted into cDNA using Superscript IV and random hexamers. Long (1.5–2.0 kb) overlapping amplicons were generated from the S and L segment using a custom-designed primer panel (Supplementary Table 2) and repliQa HiFi ToughMix (Quantabio, Beverly, MA, USA). Libraries were prepared using Illumina DNA Prep and sequenced using a NextSeq2000 sequencer (Illumina, San Diego, CA, USA). Sequence analysis and assembly were performed using a pipeline developed in house. Briefly, FASTQ files were quality-trimmed and then adapter- and primer-trimmed using BBDuk (Joint Genome Institute, Berkley, CA, USA). Trimmed FASTQ files were then aligned separately with the S and L segments using the MEM algorithm in Burrows–Wheeler Aligner v0.7.17^86^. Consensus sequences were generated using SAMtools v1.16^87^. Variant analysis was performed using Genome Analysis Toolkit (GATK) v4.4.0.0 haplotype caller^88^; GATK variant filtration was done with hard-filtering criteria for single-nucleotide polymorphisms (SNPs) and indels (Supplementary Table 3).

### Animal studies

All animal studies were approved by the National Institutes of Health (NIH) National Institute of Allergy and Infectious Diseases (NIAID) Division of Clinical Research (DCR) Institutional Animal Care and Use Committee and performed in animal BSL-4 (ABSL-4) laboratories at the IRF-Frederick, an institute fully accredited by the Association for Assessment and Accreditation of Laboratory Animal Care International (AAALAC). Domesticated guinea pigs (*Cavia porcellus* (Linnaeus, 1758)) were housed in the ABSL-4 laboratory, monitored daily for signs of disease, anesthetized using isoflurane for venipuncture of the anterior venae cavae, and exsanguinated prior to humane euthanasia when criteria were met or at study termination, followed by necropsies. Specifically, guinea pigs were anesthetized via inhalation using 4–5% isoflurane mixed in 100% oxygen to effect using an induction box. If necessary, anesthesia was maintained by continuous 2% isoflurane mixed in 100% oxygen using nose cones for the duration of the procedure. Guinea pigs with a clinical score of 3 were euthanized immediately, and all remaining animals were euthanized at study termination at 42 d post-exposure. Terminally, guinea pigs were deeply anesthetized by intraperitoneal injection of ketamine and xylazine or by inhalation of isoflurane, exsanguinated via direct cardiac puncture or by cutting of the caudal venae cavae and then euthanized by injecting an overdose of pentobarbital. Animal groups were blinded to all staff performing clinical observations and other downstream analyzes.

### Evaluation of rLASV/IGR-CD attenuation in strain 13 guinea pigs

Strain 13 guinea pigs (5–16-week-old male and female), obtained from the IRF-Frederick breeding colony, were assigned to two groups of *n* = 8. Groups were established to distribute age and weight proportionally. Animals (*n* = 8) were exposed subcutaneously to 10^5^ PFU of rLASV/IGR-CD or rLASV-WT. At 7, 14, 21, 28, 35, and 42 d post-exposure, blood was sampled from the cranial venae cavae to determine viral loads and antibody responses, as described previously^43,44^.

### rLASV/IGR-CD vaccination studies in guinea pigs

Strain 13 guinea pigs (5–16-week-old male and female), obtained from the IRF-Frederick breeding colony, were assigned to two groups of *n* = 5. Hartley guinea pigs (7–9-week-old male and female), obtained from Charles River Laboratories, Saint Constant, QC, Canada, were assigned to three groups of *n* = 8. Groups were established to distribute age and weight proportionally. Strain 13 guinea pigs were vaccinated subcutaneously with PBS or 10^5^ PFU of rLASV/IGR-CD. Hartley guinea pigs were vaccinated subcutaneously with PBS or 10^2^ PFU or 10^4^ PFU of rLASV/IGR-CD. Animals were observed daily for clinical signs for 30 d, and animal weights and temperatures were measured weekly. At 30 d, animals were exposed subcutaneously to rLASV-WT (strain 13) or intraperitoneally to GPA-LASV (Hartley). At -30, -23, -16, -9, -2, and 16 d post-exposure, blood was sampled from the cranial venae cavae to determine viral loads and antibody responses, as described previously^43,44^. Livers, spleens, kidneys, and lungs were collected at necropsy for pathology and viral-load analyzes.

### Viral load measurements

Viral supernatant samples were collected during the in vitro growth kinetics experiments, whole blood was sampled at the indicated time points and just prior to necropsy, and tissue samples were collected at necropsy. Samples were inactivated with TRIzol LS (Thermo Fisher Scientific). Total RNA was isolated using the KingFisher Sample Purification System and MagMAX Viral Pathogen Nucleic Acid Isolation Kit (Thermo Fisher Scientific). Briefly, a volume of 200 µL of inactivated sample was added to 550 µL of binding solution and magnetic beads. After binding and washing steps (with 80% ethanol [VWR, Radnor, PA, USA]), samples were eluted into 70 µL of elution buffer (Thermo Fisher Scientific). Viral loads in the sample were measured using RT-qPCR with LASV L forward primer (GACGCTAGATCGCTCATGAAT), LASV L reverse primer (TTGGAGGATAGGGTTGGTTTG), and LASV L probe (56-FAM-TCTCAAACACTGATGGGTACAGCCT-36-TAMsp). The standard curve spanned 10^8^ copies per reaction (upper limit of quantification [ULOQ]) through 10 copies per reaction (lower limit of quantification [LLOQ]). Transformed data from all samples were plotted in viral RNA copies (log_10_) per mL (blood or viral supernatant) or viral RNA copies (log_10_) per mg (tissue).

### Antibody measurements

To measure LASV-specific antibody titers, an IgG enzyme-linked immunosorbent assay (ELISA) was developed in-house. LASV antigens used in this assay were crude cell extracts generated from LASV-WT-infected Vero cells. Extracts were lysed with radioimmunoprecipitation buffer (Cell Signaling Technology, Danvers, MA, USA) and gamma-irradiated (50 kGy) to inactivate replicative virus before removal from the BSL-4 laboratory. Plates were coated with LASV-infected cell extracts diluted in coating buffer (Biolegend, San Diego, CA, USA) at a concentration of 50 ng per well, and plates were stored at 4 °C. Plates were washed six times with PBST (PBS + 0.2% TWEEN 20 [MilliporeSigma]), and a volume of 300 μL of blocking buffer (PBST + 3% normal chicken serum [Abcam, Boston, MA, USA] + 2% non-fat milk [Thermo Fisher Scientific]) was added to each well. After incubation at 37 °C for 1 h, heat-inactivated irradiated plasma that was serially diluted two-fold was added to the plates, and plates were kept at 4°C overnight. After washing the plates six times with PBST, goat anti-guinea-pig IgG horseradish peroxidase (MilliporeSigma) was added. The plates were incubated at 37 °C for 1 h and washed again with PBST. Antibody-antigen complexes were revealed by adding 3,3ʹ,5,5ʹ-tetramethylbenzidine substrate (Thermo Fisher Scientific) and incubating for 10 min at room temperature, and the reaction was stopped with stop solution. The absorbance was read at 450 nm on an Infinite M1000 plate reader (Tecan, Morrisville, NC, USA). The average signal from normal guinea pig plasma plus 3× standard deviations was set as the cutoff value for endpoint titer measurement. Reciprocal serum dilutions corresponding to minimal binding were used to calculate titers.

### Host chemokine/cytokine response assays

Host chemokines and cytokines were measured on a Luminex MAGPIX Multiplexing System (Luminex Corporation, Austin, TX, USA). Analytes in first panel (ID.MCTOMAG-70K-06) included CXCL2 (MIP-2), CXCL5(LIX), CXCL10 (IP-10), IL12B (IL12p40), IL13, and VEGFA. The second panel (ID.MTH17MAG-47K-10) measured IFNG, IFNL3 (IL-28B), IL5, IL12 (IL12p70), IL13, IL17A, IL22 (IL17E), IL23, IL25, and IL33. Briefly, sera isolated from blood collected from virus-exposed strain 13 guinea pigs were added to magnetic beads conjugated to antibodies that target the indicated analytes. After a series of washes, secondary antibodies conjugated to phycoerythrin were added to the magnetic beads and loaded into the MAGPIX instrument for analysis with the appropriate kits. Raw data were exported as CSV files and analyzed in Excel using Bioplex Results Generator 3.0 and Bioplex Manager 6.1. Concentrations of each analyte were based on the standard curve generated from standards provided by the manufacturer.

### Histopathology, immunohistochemical, and in situ hybridization studies

Guinea pigs were humanely euthanized at the end of each experiment and complete necropsies were performed. All major organs were collected and fixed in 10% neutral-buffered formalin for at least 72 h in biosafety level 4 (BSL-4) containment before routine processing in a Tissue-Tek VIP-6 vacuum infiltration tissue processor (Sakura Finetek USA, Torrance, CA, USA), and paraffin-embedded via a Tissue-Tek TEC-6 embedding station with cryo module (Sakura Finetek USA, Torrance, CA). Paraffin-embedded tissues were sectioned at 4 µm using a standard semiautomated rotary microtome Leica RM2255 (Leica Biosystems, Buffalo Grove, IL, USA); mounted on positively charged glass slides; air-dried for routine hematoxylin and eosin (H&E) staining, IHC, or RNAscope in situ hybridization (ISH); and coverslipping with a nonalcohol-based mounting medium. IHC was performed with an anti-LASV-NP monoclonal antibody (Cambridge Biologics, Brookline, MA, USA; #01-04-0104), followed by a biotinylated secondary antibody and an avidin/biotin based tertiary antibody. RNAscope ISH was performed to detect LASV genomic RNA in formalin-fixed, routinely processed, paraffin-embedded, 4-µm sliced, mounted tissue sections on charged glass slides, using the manual RNAscope 2.5 HD RED Detection Kit (Advanced Cell Diagnostics, Newark, CA) in accordance with the manufacturer’s protocol, including modifications for optimization validated by appropriate controls. The probe pairs used targeted the LASV genomic *Z* and *L* genes (catalog no. 463761, ACD). Slides were examined by a pathologist and imaged as described previously^43,44^.

### Isolation of splenocytes, cell banking, and thawing

Spleens were weighed, homogenized in c-tubes by Gentle MACS dissociation (Miltenyi, Gaithersburg, MD, USA), washed with 2% FBS PBS + 2 mM EDTA (PBS-2), strained through a 100-µM filter, ACK-lysed, and counted on a cell counter (Nexcelom, Lawrence, MA, USA). Isolated cells were washed with PBS-2, resuspended to approximately 5–30 × 10^6^ live cells per mL in Recovery Cell Culture Freezing Medium (Thermo Fisher Scientific), aliquoted into Cryovials (Thermo Fisher Scientific), initially cryopreserved in a −80 °C freezer, and subsequently moved to long-term storage in a −150 °C freezer within 48 h.

Frozen splenocytes were thawed in a similar manner to procedures previously described^89^. In brief, frozen splenocyte cryovials were inverted and placed within CryoThaw Tube Adapters (Medax International, Salt Lake City, UT, USA) on 15-mL conical tubes containing 9 mL of PBS-2. Then, samples were washed with PBS-2 and resuspended in 1 mL of PBS-2. Thawed cells were counted, and up to 2.5 × 10^6^ live splenocytes were aliquoted per sample for flow cytometry staining.

### Antibody conjugation for flow cytometry panel

Unconjugated antibodies specific for guinea pig markers (Bio-Rad, Hercules, CA, USA) were conjugated at the IRF-Frederick to fluorescent dyes in accordance with company-provided protocols using the respective Lightning Link Fast Conjugation Kits (Abcam, Cambridge, UK) for the following antibodies of interest: rabbit anti-guinea-pig IgG, APC; mouse anti-guinea-pig T lymphocytes, APC-Cy7; mouse anti-guinea-pig CD8, PE-Cy5; mouse anti-guinea-pig CD1b3, PE-Cy7; and mouse anti-guinea-pig MHC Class II, PerCP-Cy5.5. A fraction of each conjugated antibody was tested by incubation with Invitrogen UltraComp eBeads (Thermo Fisher Scientific) to ensure proper antibody: fluorophore conjugation. Prior to staining study-related samples, all antibodies were titrated on banked PBMCs isolated from guinea pig whole blood (Biochemed Services, Winchester, VA, USA). The respective conjugated antibodies were each aliquoted and stored per company provided protocols.

### Flow cytometry staining and acquisition

Up to 2.5e6 isolated cells from spleen tissue samples were resuspended to a volume of 100 µL and blocked with 10 µL of normal guinea pig serum (Jackson ImmunoResearch, West Grove, PA, USA) for 10 min on ice to inhibit Fc receptor-specific binding. Blocked samples were stained with a cocktail of primary antibodies (CD45 FITC, CD4 PE, IgG APC, T-lymphocytes APC-Cy7, CD8 PE-Cy5, CD1b3 PE-Cy7, MHC-II PerCP-Cy5.5, and CD14 BV570) and amine-reactive dye Live Dead Blue (Thermo Fisher Scientific) for dead cell exclusion and then diluted in Brilliant Stain Buffer Plus (BD Biosciences, Franklin Lakes, NJ, USA) for 20–30 min on ice. Samples were washed, fixed, and inactivated for at least 30 min with a minimum of 500 µL of Cytofix/Cytoperm (BD Biosciences), resuspended in PBS, and run on a five-laser Aurora Flow Cytometer (Cytek Biosciences, Fremont, CA, USA) on low or medium flow rate settings. Flow data was analyzed in FlowJo software version 10.8.1.

### Statistical analysis

The log-rank (Mantel–Cox) test was used for survival curve comparison. Statistically significant differences in animal weights/temps, ELISA endpoints, cytokine concentrations, and virus titers were determined by unpaired Student’s t-test. Statistically significant differences in cellular frequencies from the flow cytometry data were determined by grouped two-way ANOVA with multiple comparisons. (*p < 0.05, significant; **p < 0.01, very significant; ***p < 0.001, highly significant; n.s., *p* > 0.05, not significant). Prism 9 (GraphPad Software) was used for all statistical analyzes.

## Supplementary information

Supplementary information

## Data Availability

All relevant data are available from the corresponding author upon reasonable request. Sequence data have been deposited in the NCBI Sequence Read Archive (SRA) under BioProject PRJNA1168054. The genomic large and small segment sequences of rLASV/IGD-CD have been deposited to GenBank under accession numbers PQ421570 and PQ421571, respectively.
