# Supplementary material for: A Lassa virus live attenuated vaccine candidate that is safe and efficacious in guinea pigs
Source: NPJ Vaccines. 2024 Nov 17;9:220. doi: 10.1038/s41541-024-01012-w (PMC11570604; doi:10.1038/s41541-024-01012-w)
Supplement: Supplementary file 1 — Supplementary information [file 41541_2024_1012_MOESM1_ESM.pdf]

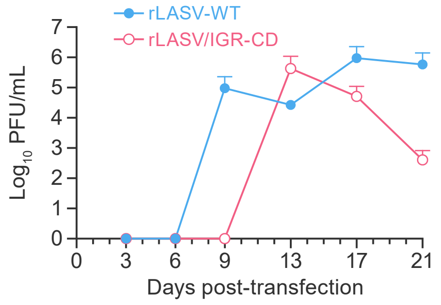

Supplementary Figure 1: Generation and characterization of rLASV/IGR-CD in cultured cells. Expression plasmids encoding LASV NP, LASV L, and T7 RNA polymerase were transfected into HEK293T cells along with plasmids containing the antigenomic S and L segments of LASV under the control of the T7 promoter to create rLASV-WT, or the rLASV-GPC/CD S and rLASV/IGR(S-S) L segments under the control of the T7 promoter to create rLASV/IGR-CD. At Day 3 post-transfection, Vero cells were added to the transfected HEK293T cells at a 1:1 ratio and incubated for an additional 18 d. Tissue-culture supernatant (TCS) samples were collected at the indicated time points, and plaque assays were performed to determine virus titers. *n*=4, error bars indicate standard deviation. CD, codon deoptimized; HEK293, human embryonic kidney epithelial 293T/17 cells; IGR, intergenic region; L, large (protein/segment); LASV, Lassa virus; NP, nucleoprotein; PFU, plaque-forming units; rLASV, recombinant LASV; S, small segment; WT, wild-type.

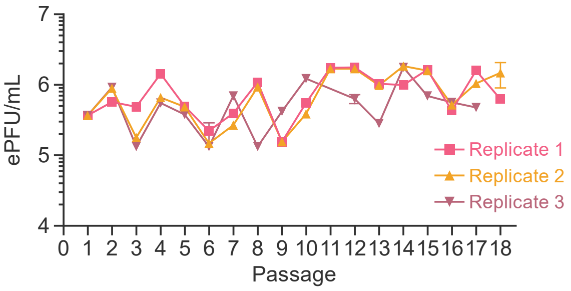

Supplementary Figure 2: Assessment of rLASV/IGR-CD stability during serial passages in cultured cells. Vero cells were exposed (MOI=0.01) to rLASV/IGR-CD and, at 72 h post-infection, cell-culture supernatants were collected and used to initiate serial passages (total of 18) in Vero cells. Samples were collected at each passage and levels of LASV RNA assessed by RT-qPCR. Results were converted to equivalent PFU/mL (ePFU/mL) which was used to determine the volume of virus needed to infect for the next passage. CD, codon deoptimized; ePFU, equivalent plaque-forming units; IGR, intergenic region; LASV, Lassa virus; MOI, multiplicity of infection; rLASV, recombinant LASV; CD, codon-deoptimized; RT-qPCR, real-time reverse transcription polymerase chain reaction.

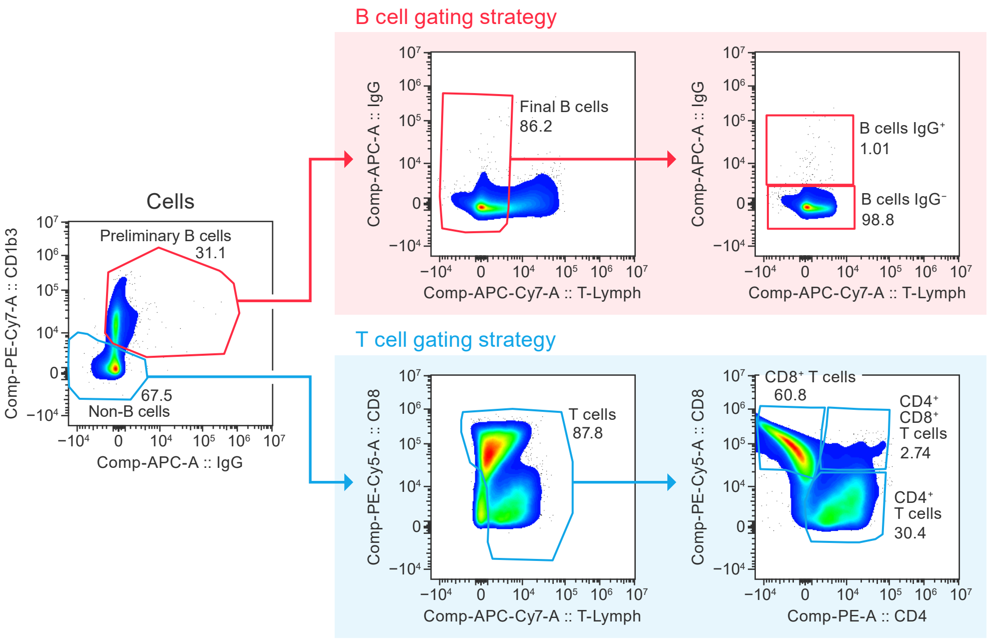

Supplementary Figure 3: Evaluation of rLASV/IGR-CD induced immunity in strain 13 guinea pigs. Flow cytometry gating strategy for splenocytes. Cells isolated from spleens were stained with a newly developed flow cytometry panel and run on a flow cytometer to identify distinct populations of splenic immune cells. A gate was denoted for time to exclude the events collected for the first few seconds. Samples were further gated for singlets based on FSC (FSC-A vs. FSC-H) and SSC (SSC-A vs. SSC-H) parameters. Singlets were further gated on lymphocytes (FSC-A^{low}, CD14^{-}), and live CD45^{+} lymphocytes. Cells were further differentiated into preliminary B Cells (CD1b3^{+}, IgG$^{+/-}$) or non-B cells (CD1b3^{-}, IgG^{-}). CD1b3^{+} B cells were further gated on the lack of staining for the T-lymphocyte marker (IgG$^{+/-}$, T-lymphocyte^{-}). Final B cells were last separated on the presence or absence of IgG denoting class switching to IgG. Non-B cells were gated on the expression of T-lymphocyte marker (T-lymphocyte^{+}, CD8$^{+/-}$), and T cells were subsequently separated based on staining of CD8 (CD8^{+} T cells), CD4 (CD4^{+} T cells), or co-staining of CD4 and CD8 (CD4^{+} CD8^{+} T cells). CD, cluster of differentiation; FSC, forward scatter; SSC, side scatter.

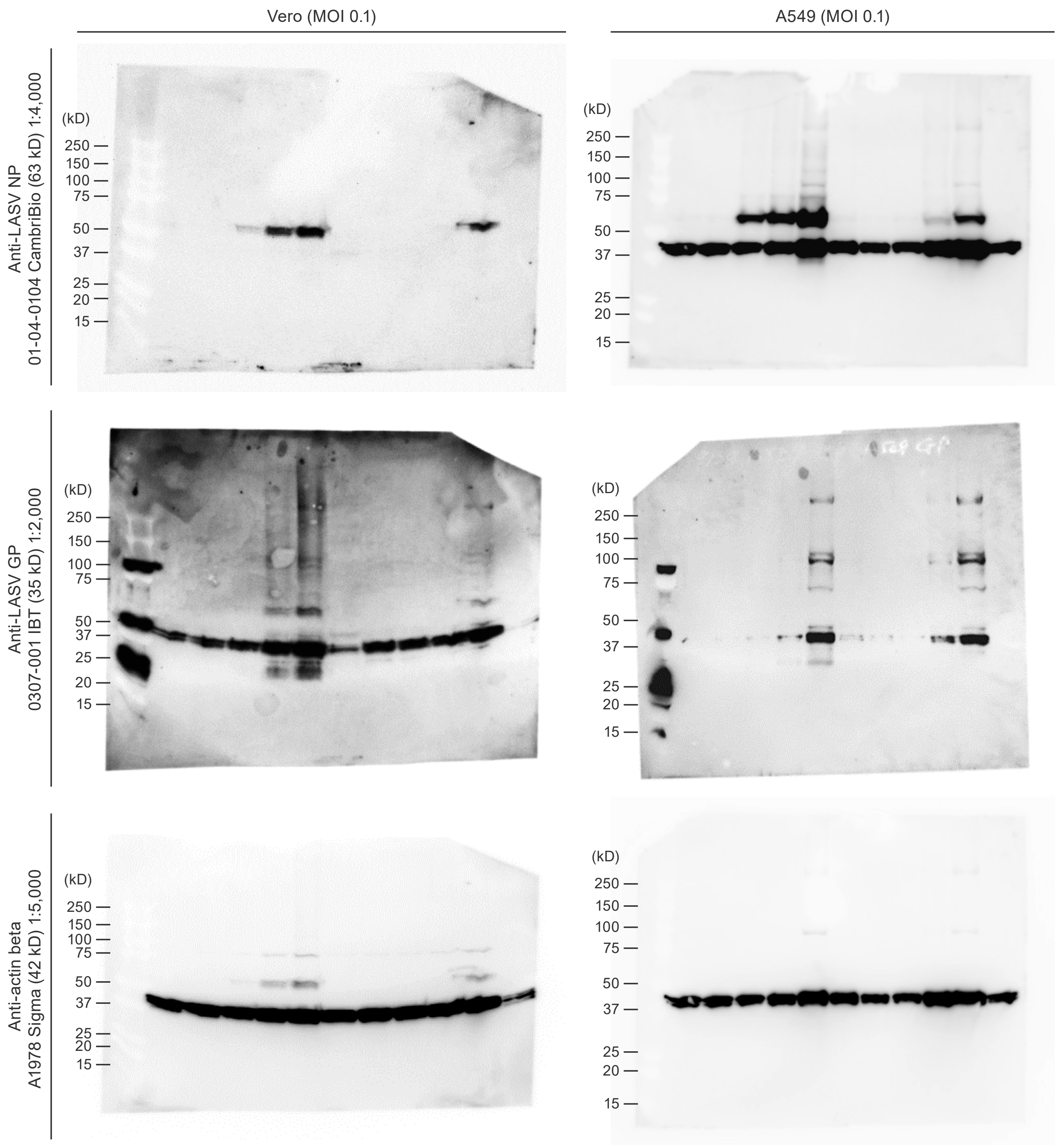

Supplementary Figure 4: Uncropped and unprocessed scans of western blots shown in Figure 2A.

Supplementary Table 1: Assessment of rLASV/IGR-CD stability during serial passage in cultured Vero cells.
Mutations incorporated into the rLASV/IGR-CD genome during serial passages in Vero cells.

L Segment											
POS	REF	ALT	P13R1	P13R2	P13R3	P18R1	P18R2	P18R3	Codon change	AA change	Protein
**812**	**G**	**A**						**99, 39**	**GCC to ACC**	**A 271 to T**	**L**
**1550**	**T**	**C**		**40, 99**			**17, 57**		**TCC to CCC**	**S 517 to P**	**L**
1657	C	T		69, 98			23, 54		TCC to TCT	S 552 to S	L
**2100**	**C**	**T**		**830, 599**			**279, 511**		**GCT to GTT**	**A 700 to V**	**L**
2545	T	C				502, 597			GGT to GGC	G 848 to G	L
**3042**	**A**	**G**			**1109, 398**				**AAG to AGG**	**K 1014 to R**	**L**
**3062**	**A**	**G**						**902, 337**	**ATG to GTG**	**M 1021 to V**	**L**
**3064**	**G**	**A**		**74, 1400**		**835, 476**	**0, 604**	**822, 426**	**ATG to ATA**	**M 1021 to I**	**L**
4114	T	C				572, 597			AGT to AGC	S 1371 to S	L
**4521**	**A**	**T**	**1174, 486**		**876, 363**				**TAC to TTC**	**Y 1507 to F**	**L**
5134	T	C			1108, 351				AAT to AAC	N 1711 to N	L
6742	T	C				618, 761			AGT to AGC	S 2247 to S	L
6763	G	A				608, 755			AAG to AAA	K 2254 to K	L
**6837**	**C**	**G**				**569, 673**				**None**	**IGR**
**6862**	**CG**	**C**					**627, 255**			**None**	**IGR**
**6964**	**G**	**T**		**1293, 1424**			**778, 923**	**1436, 532**	**ACA to AAA**	**T 95 to K**	**Z**

S Segment											
POS	REF	ALT	P13R1	P13R2	P13R3	P18R1	P18R2	P18R3	Codon change	AA change	Protein
**50**	**T**	**C**				**508, 1600**			**TTT to TCT**	**None**	**5' UTR**
**428**	**A**	**G**	**1201, 5439**	**2822, 3707**	**2617, 4000**	**954, 5625**	**988, 5672**	**4486, 1941**	**AAA to AGA**	**K 143 to R**	**GPC**
1275	G	A	1368, 5313			200, 6696			GCG to GCA	A 425 to A	GPC
**2125**	**T**	**C**		**119, 6334**		**217, 5958**	**0, 6448**	**783, 5526**	**AGA to GGA**	**R 426 to G**	**NP**
2150	A	G		4922, 1757				4970, 1627	GCT to GCC	A 417 to A	NP
**2740**	**T**	**C**		**2682, 4387**			**31, 6971**	**4615, 2235**	**ACA to GCA**	**T 221 to A**	**NP**

Sequencing results from serial passaging of rLASV/IGR-CD showing both synonymous and non-synonymous mutations. The first number in every box shows the allelic depth for the reference allele, while the second number indicates allelic depth of the alternate allele. Empty boxes indicate that no variant allele was seen at that position. Rows with bold letters and numbers indicate non-synonymous mutations, and rows with no bold letters or numbers indicate synonymous mutations.

Supplementary Table 2: Assessment of rLASV/IGR-CD stability during serial passage in cultured Vero cells.

Primer Name	5'-Sequence-3'	
86_LASV_Lpan_Fwd1	TTGTGCGCTGTACTTCTCCAAA	Large seg Pool 1. Start: 41
87_LASV_Lpan_Rev1	GAACTGGGTGCATGGGTCTTAG	Large seg Pool 1. Start: 2022
88_LASV_Lpan_Fwd2	TTTGGTTGACGCTAGATCGCTC	Large seg Pool 2. Start: 1803
89_LASV_Lpan_Rev2	ATAGCCAAGAGTCGCCACAATC	Large seg Pool 2. Start: 3824
90_LASV_Lpan_Fwd3	TCCTTCTCGTTGTTCAGGCAAC	Large seg Pool 1. Start: 3616
91_LASV_Lpan_Rev3	CTTTCCTCATTGAGCCTCGCTT	Large seg Pool 1. Start:5631
92_LASV_Lpan_Fwd4	GAGAACGTTTACTGGGGTGAGC	Large seg Pool 2. Start: 5219
93_LASV_Lpan_Rev4	CTTGTGTCTTCTGTGCCCCTAC	Large seg Pool 2. Start: 7227
339_LASV_S_472_M13_2kb_Fwd	GTAAAACGACGGCCAGTGGGGATCCTAGGCATTTTTGGT	Small seg Pool 1. Start: 6
340_LASV_S_472_M13_2kb_Rev	GTAAAACGACGGCCAGTTCAACCAAGTTCAGGCTGCTAC	Small seg Pool 1. Start: 2093
343_LASV_S_472_M13_1.5kb_Fwd	GTAAAACGACGGCCAGTTTCCTTATGTCATCGGACCCCT	Small seg Pool 2. Start: 1895
342_LASV_S_472_M13_2kb_Rev	GTAAAACGACGGCCAGTCCTAGGCTATTGGATTGCGCTT	Small seg Pool 2. Start: 3389

Supplementary Table 3: Genetic stability assessment. Hard filter criteria for Genome Analysis Toolkit (GATK) variant filtration.

Type	Read Depth	Quality By Depth (QD)	Fisher Strand (FS)	Mapping Quality (MQ)	Read Position Rank Sum	Strand Odds Ratio
SNPs	<20.0	<5.0	>60.0	<40.0	<−8.0	>3.0
Indels	<20.0	<5.0	>200.0	N/A	N/A	>10.0

SNP, single nucleotide polymorphism; indel, small insertion or deletion; FisherStrand, Strand bias estimated using Fisher's exact test; N/A, not applicable.
